# Scaling Pattern to Variations in Size during Development of the Vertebrate Neural Tube

**DOI:** 10.1016/j.devcel.2016.03.024

**Published:** 2016-04-18

**Authors:** Aysu Uygur, John Young, Tyler R. Huycke, Mervenaz Koska, James Briscoe, Clifford J. Tabin

**Affiliations:** 1 Department of Genetics, Harvard Medical School, 77 Avenue Louis Pasteur, Boston, MA 02115, USA; 2 Mill Hill Laboratory, The Francis Crick Institute, London NW7 1AA, UK

## Abstract

Anatomical proportions are robustly maintained in individuals that vary enormously in size, both within a species and between members of related taxa. However, the mechanisms underlying scaling are still poorly understood. We have examined this phenomenon in the context of the patterning of the ventral neural tube in response to a gradient of the morphogen Sonic hedgehog (SHH) in the chick and zebra finch, two species that differ in size during the time of neural tube patterning. We find that scaling is achieved, at least in part, by altering the sensitivity of the target cells to SHH and appears to be achieved by modulating the ratio of the repressive and activating transcriptional regulators, GLI2 and GLI3. This mechanism contrasts with previous experimental and theoretical analyses of morphogenic scaling that have focused on compensatory changes in the morphogen gradient itself.

## INTRODUCTION

The bodies of animals from a particular taxa share a common “baupläne” or blueprint. Fundamental baupläne of phylum-level groups are often modified in size and proportion and through fusions, duplications, or losses, yet are nonetheless recognizable as an underlying principle in all derived forms. Among more closely related taxa and in individuals of the same taxa, there is a more conservative adherence to a common baupla¨ne. Not only are the same structures present, but they are often maintained in relative proportion, a phenomenon known as scaling.

Scaling can be achieved at several different developmental stages (Barkai and Ben-Zvi, 2009; Umulis and Othmer, 2013). For example, an initial pattern can be established when an embryo is a set size and dimension, followed by differential yet proportional growth. However, in many instances embryos of related taxa are of quite different size at the time patterning is established, although the ultimate proportion of anatomical and cellular structures are nonetheless scaled. In such instances the patterning mechanisms themselves must be modified to generate a size-invariant output. For example, one classic patterning mechanism is the morphogen gradient, in which a signal (the “morphogen”) is secreted from a “signaling center” at one end of a developmental field. The morphogen becomes more dilute as it spreads away from the source and the target tissue responds by activating distinct transcriptional programs in a concentration-dependent manner, thereby establishing distinct cell fates at specific morphogen concentration thresholds (Lander, 2007). While this is a simplified description of a morphogen-based patterning mechanism, it serves to illustrate the problem faced by developmental systems in scaling patterns. How can such a morphogen system be adjusted to trigger the same transcriptional responses, in proportional domains, across a smaller developmental field and/or when less morphogen is produced from a smaller signaling center?

We have explored this question in the context of the developing neural tube. The ventral neural tube is one of the best-understood examples of patterning in response to the gradient of a morphogen. In this case, the morphogen is the secreted protein Sonic hedgehog (SHH). During neural tube development, SHH is secreted from the subadjacent ventral notochord and floor plate (Jessell, 2000). However, SHH expression is initiated first in the notochord, and progenitor patterning is largely dependent upon notochord-derived SHH (Chamberlain et al., 2008; Yu et al., 2013). As SHH protein diffuses dorsally, the resulting gradient regulates the expression of a series of transcription factors at threshold concentrations, thereby establishing molecularly distinct domains of progenitors, each of which ultimately gives rise to different neuronal subtypes. The pattern of cellular differentiation is dictated by both amount and duration of exposure to the morphogen (Dessaud et al., 2007). While the transcription factors activated by SHH activity are themselves responsible for determining neural cell fate, in a practical sense they can also be used as markers in vitro and in vivo, as readout of the various threshold responses to the SHH gradient. Thus, OLIG2 expression marks motor neuron progenitors (pMN) (Mizuguchi et al., 2001; Novitch et al., 2001), NKX2.2 expression marks the more ventral v3 interneuron progenitors (p3), and NKX6.1 is expressed in three ventral progenitor domains (pMN, p3, and p2) (Briscoe et al., 2000). In contrast, increasing concentration or duration of SHH signaling represses expression of PAX7, a transcription factor expressed in dorsal progenitor domains in the neural tube, and of PAX6, whose expression is increasingly restricted dorsally as patterning progresses in vivo (Ericson et al., 1997). These markers for different levels of SHH signaling in the developing neural tube provide a unique opportunity to assess how morphogen patterning is scaled in a vertebrate context.

Although scaling of SHH, in particular, has not previously been examined, the scaling of other morphogens has been examined both experimentally and at a theoretical level. These studies have revealed several ways whereby gradients can be scaled to embryo size. Moderate changes in tissue size can be accommodated by proportional changes in the amount of morphogen production (Cheung et al., 2014). Gradients can be scaled over larger size differences by changes in ligand diffusion and/or decay rate, as seen in the scaling of the bicoid gradient between different *Drosophila* species (Gregor et al., 2005). This can be achieved by feedback mechanisms in which the expression of an “expander” molecule changes the diffusion or decay rate (Ben-Zvi and Barkai, 2010). Alternatively, scaling can be achieved using two opposing gradients (Houchmandzadeh et al., 2005; Howard and ten Wolde, 2005; McHale et al., 2006). This requires cells to compare relative amounts of the two morphogens and for the external gradients to change in concert with one another. In principle, such a system could be at play in the neural tube, as it is known that morphogens of the bone morphogenetic protein (BMP) family, produced in the surface ectoderm and roof plate to specify dorsal cell types (Liem et al., 1997), act in opposition to the ventral SHH gradient (Liem et al., 2000). What each of these mechanisms has in common is that they rely on changes external to the target cells to reshape the ligand gradient. An alternative, which has not previously been described, would be to scale patterning in a cell-autonomous manner by altering the intrinsic sensitivity of the target cells to the morphogen. This, indeed, appears to be the case regarding the morphogen scaling investigated here.

## RESULTS

### The Neural Tubes of the Chick and Zebra Finch Are Proportionally Scaled

**Figure 1:**
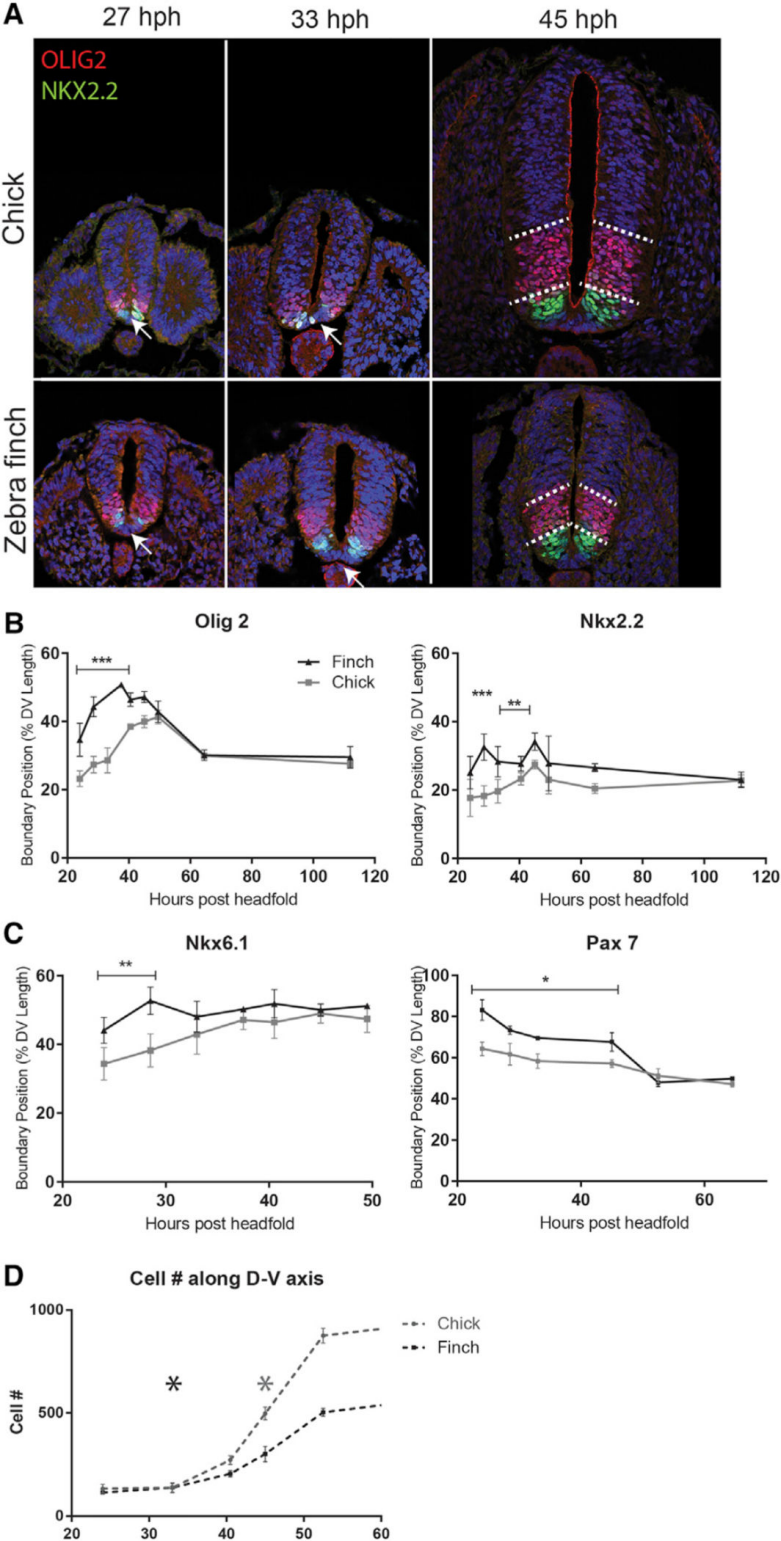
Although Ultimately Scaled, Progenitors Are Patterned More Rapidly in Smaller Birds (A) Chick and zebra finch neural tubes at different stages of development, 27, 33, and 45 hr post headfold (hph). At stages 27 and 33 hph the dorsal expansion of transcription factors OLIG2 and NKX2.2 expression, which is induced by SHH morphogen activity, appears more advanced in the zebra finch neural tube compared with the chick neural tube. Other hallmarks of the patterning process, such as exclusion of NKX2.2 from floor plate, are also achieved faster in the zebra finch (arrows). At 45 hph, patterning is set for both species, and scaled accordingly despite the difference in size. Scale bar, 100 µm. (B) Dorsal expansion of OLIG2 and NKX2.2 is plotted for chick and zebra finch neural tubes for a range of developmental stages. As was shown in (A), the final position of the dorsal boundary along the dorsoventral axis is achieved more rapidly for both markers in the zebra finch, even though ultimate proportions are scaled to size before onset of differentiation. Error bars denote SD. **p < 0.01, **p < 0.001. (C) Dorsoventral patterning is accelerated for other transcription factors. NKX6.1, whose expression is induced by SHH activity, completes its dorsal expansion at an earlier developmental stage in the zebra finch neural tube. PAX7, whose expression is suppressed by SHH activity, is initially restricted more dorsally in the finch neural tube. Eventually PAX7 expression is confined to the dorsal 50% of the neural tube in both species. Error bars denote SD. *p < 0.05, **p < 0.01. (D) Cell number along the dorsoventral axis of the zebra finch versus chick neural tube is comparable at the earliest stages of development. However, the difference in size escalates as chick neural tube grows to roughly twice the size of finch neural tube. Patterning is still dynamic at the time of this size difference, as patterning of progenitors appears to be complete in chick ~45 hph (gray asterisk), and in zebra finch ~30 hph (black asterisk). Error bars denote SD.

To explore the mechanisms underlying scaling of size and pattern in the developing ventral neural tube, we chose two avian species that are strikingly different in size from a very early time in development, the zebra finch (*Taeniopygia guttata*) and the chick (*Gallus gallus*). To first ascertain whether dorsoventral patterning along the neural tubes of these differently sized birds is indeed scaled, we took advantage of the well-established set of transcription factors that are expressed in response to different levels of SHH activity in the developing neural tube, including (from ventral to dorsal) NKX2.2, OLIG2, and NKX6.1. We collected neural tube tissue adjacent to somite 15 (forelimb level for both chick and zebra finch) across a series of developmental stages. Indeed, both species end up with the dorsoventral length of progenitor domains proportionally scaled to the final size of the neural tube, prior to the onset of neural differentiation (Figure 1A). Both the relative dorsoventral length and relative cell number of these domains are proportional (Figure S1A).

One obvious mechanism that could explain scaling of the neural tube would be for all birds to pattern the neural tube at stages when the dorsoventral axis of this structure is of a standard size followed by proportional expansion to different extents in distinct species. Therefore, we next determined the developmental time when the neural tube is patterned in each species based on when the molecularly defined progenitor pools achieve their final proportions. Strikingly, for all transcription factor markers analyzed, patterning is accelerated in the smaller zebra finch (Figures 1A–C and S1A).The final proportions of the ventral domains are established by approximately 45 hr post headfold (hph) in the chick, but by 33 hph in the zebra finch.

We next examined the growth of the neural tube during this period. For a brief time at the early stages of patterning, neural tube size is comparable between the two species. However, the dorsoventral axis of the chick neural tube grows significantly faster than the zebra finch (Figures 1D and S1B). By the time patterning is complete in the chick (~45 hph), its neural dorsoventral axis is 3.5 times the size of that of the zebra finch at the time it completes the patterning phase (~30 hph). Thus, the patterning of the zebra finch neural tube occurs faster and across a tissue of a smaller size than in the chick.

**Figure 2:**
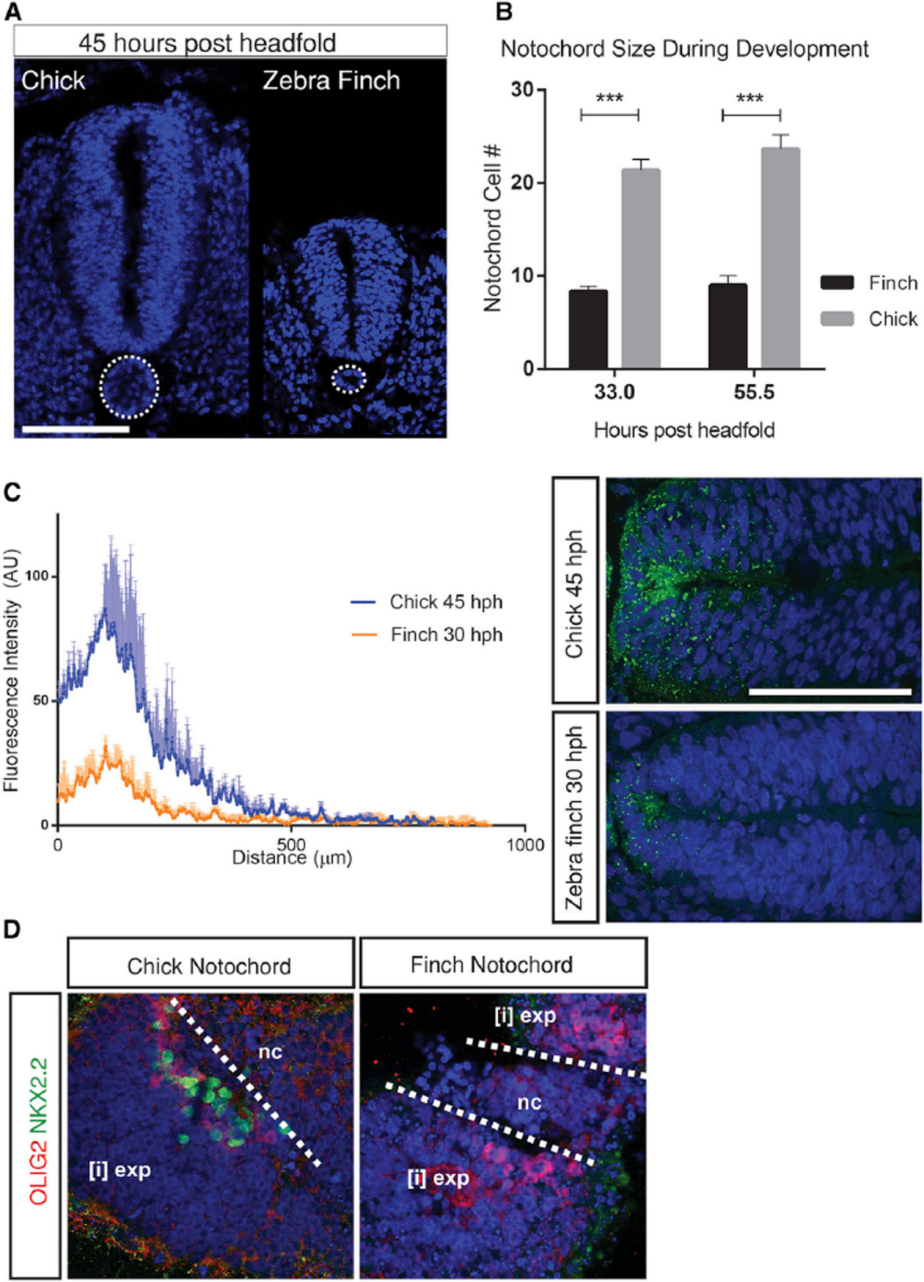
SHH Morphogen Production Is Lower in Zebra Finch than in Chick (A and B) Throughout development, both the area and number of cells in a cross section of the notochord are significantly less in the zebra finch than the chick (***p < 0.001). Notochord in the two species is outlined in white in (A). Error bars denote SD in (B). Scale bar, 100 µm. (C) To quantify and compare the amplitude and shape of the gradient in two species, SHH protein was detected in histological sections of chick and zebra finch neural tubes with the 5 ×10^1^ antibody (right panel). Fluorescence intensity of 5 ×10^1^ SHH antibody was plotted against distance along the dorsoventral axis. At stage HH 17 (when patterning is complete in chick), the amplitude of signal along the chick neural tube is markedly greater than signal amplitude at stage HH 14 in zebra finch (when patterning is complete in finch) (left panel). Error bars (upper trace only) denote SD. (D) To test whether the difference in neural tube size and gradient amplitude translate to a difference in morphogen activity, we incubated chick naive intermediate neural tube [i] explants (exp) (isolated from neural tube at stage HH 10–11) in vitro, embedded in collagen adjacent to a given length of notochord (nc) from either chick or zebra finch embryos (white dotted lines mark boundaries). Chick notochord was able to induce a more ventral response. Both OLIG2 and NKX2.2 (a transcription factor that requires higher SHH concentrations) were induced by chick notochords whereas in ex-plants incubated with zebra finch notochord, only OLIG2 expression was induced.

If the chick and zebra finch notochord produced the same amount of SHH and the resulting gradients were not modified in some way, one would expect ventral cell type specification to be carried out at similar absolute levels, resulting in the absence of scaling. However, the notochord is significantly smaller in the zebra finch than in the chick, even in relative terms (Figures 2A and 2B). Consistent with this, the amplitude of the SHH gradient is significantly smaller in zebra finch by the time patterning is complete (45 hph in the chick and 33 hph in the zebra finch) (Figure 2C).

To test whether the difference in levels is also reflected in a difference in morphogen activity, we embedded naive chick intermediate neural plate tissue ([i] explants) adjacent to either chick or zebra finch notochords (of the same length) (Figure 2D). After 24 hr of ex vivo culture, [i] explants were assayed for expression of NKX2.2 (green) and OLIG2 (red), representative of genes requiring higher and lower levels of SHH, respectively (Dessaud et al., 2008). The chick notochord induced robust expression of both NKX2.2 and OLIG2. By contrast, the zebra finch notochord induced OLIG2 expression but not NKX2.2 in [i] explants. This is consistent with the zebra finch notochord producing less SHH than its chick counterpart. Taken together, these results suggest that the zebra finch neural tube is patterned more rapidly and in response to lower levels of SHH than in the chick.

### A Cell-Autonomous Difference in Morphogen Sensitivity between Chick and Zebra Finch Neural Tissue

**Figure 3:**
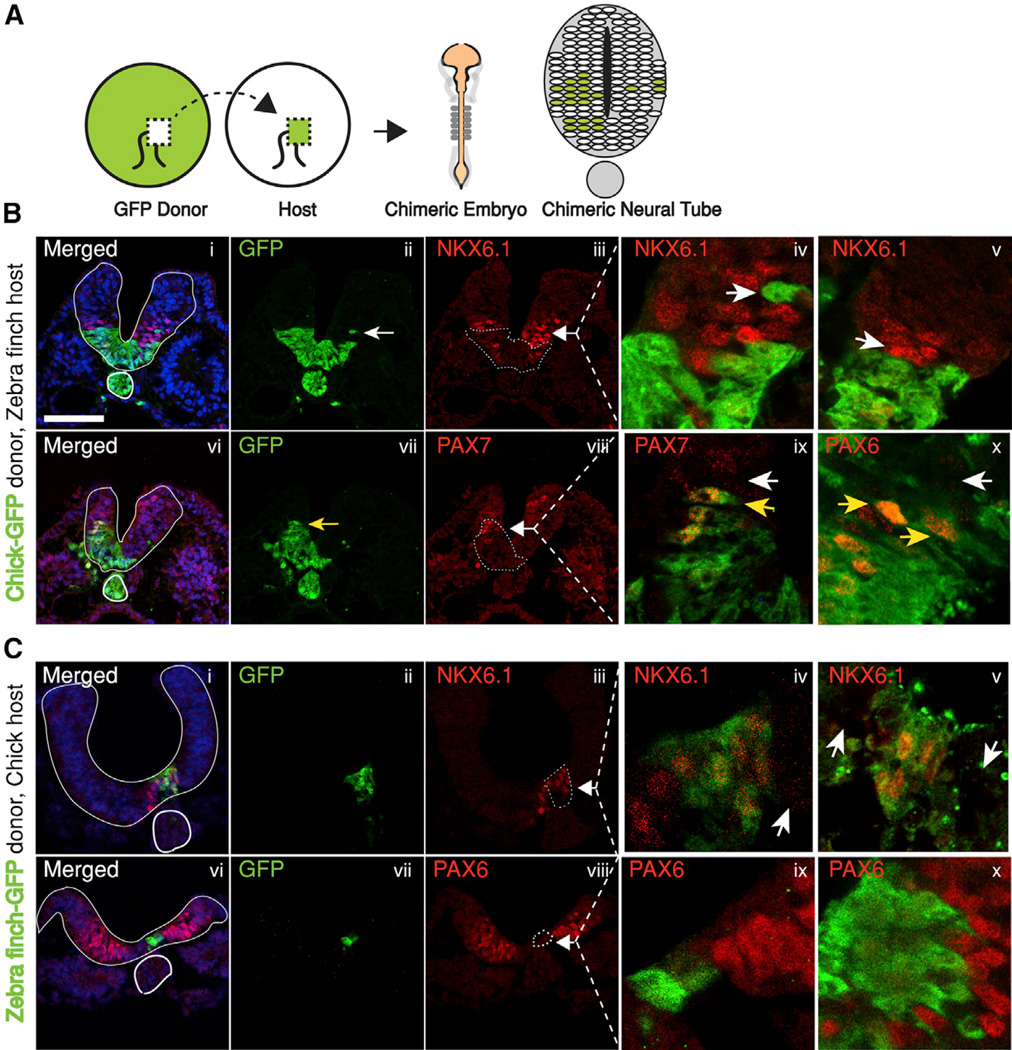
Zebra Finch Cells in Chimeric Embryos Are Cell Autonomously More Sensitive to SHH (A) After 12 hr of embryonic development (stage HH 3), perinodal tissue is transplanted from GFP donor embryo (of one species) to a non-GFP host (of the other species). Developing chimeric embryos have neural tubes with cells from both species. (B) Chimeric neural tube with GFP chick donor and non-GFP finch host. Clones of cells, as well as single cells (arrows) that express GFP are derived from the donor chick, while the rest of the neural tube is from the host zebra finch. Expression of *NKX6.1* is upregulated in zebra finch cells (panels i–v), whereas GFP^+^ chick cells at the same dorsoventral distance away from source have not yet upregulated *NKX6.1*. As shown in panel (iii) (and close-up in panel iv), a single GFP^+^ chick cell is not expressing NKX6.1, in contrast with surrounding finch cells, which have upregulated NKX6.1. Panel (v) shows a close-up of a different embryo: GFP^+^ chick cells that are more ventral (and therefore closer to the source of SHH) have not yet upregulated NKX6.1, while the more dorsal non-GFP finch cells have. Conversely for *PAX7*, finch cells in the chimeric embryo have suppressed *PAX7* in the intermediate region upon SHH exposure, whereas chick cells in the same embryo retain expression (panels vi–x). PAX7 expression in GFP^+^ chick cells is strong (yellow arrow) while it is not detected in the more dorsal finch cells (white arrows, close-up panel ix). Panel (x) shows a close-up of a different chimeric embryo stained for PAX6, another gene whose expression is suppressed by SHH and is strong in GFP^+^ chick cells, while not detected in non-GFP finch cells. n = 8/8. (C) Reciprocal experiment with finch GFP donors and chick hosts lead to similar results. GFP^+^ finch cells are more sensitive to morphogen and express NKX6.1 even when they are further away from source of SHH (panels i–iii, close-up panel iv). Chick host cells that are equidistant from source do not upregulate expression (panel iv, white arrow). Close-up of a different chimeric embryo in panel (v) shows NKX6.1 expression on the GFP^+^ finch clone only, while chick cells dorsal and ventral have not upregulated NKX6.1 (white arrows). Similarly, expression of PAX6 is suppressed in the GFP^+^ finch cells (panels vi–x), while it is still present in non-GFP chick cells. n = 4/4.

The most parsimonious explanation for a more rapid response at a lower concentration of morphogen would be that the zebra finch cells are simply more sensitive to SHH. To test whether intrinsic changes in cell sensitivity to SHH might contribute to scaling between the chick and zebra finch, we took advantage of transgenic lines of both species ubiquitously expressing GFP to create chimeric neural tubes where chick and zebra finch cells are juxtaposed in the same neural tube environment and hence exposed to identical graded SHH signals. In reciprocal experiments, perinodal tissue was transplanted from GFP embryos of one species into wild-type embryos of the opposite species at Hamburger-Hamilton (HH) stage 3 (~12 hr of incubation) (Hamburger and Hamilton, 1992). The resultant embryos had chimeric neural tubes that included GFP-expressing cells from the chick donor mixed with the non-GFP zebra finch cells or vice versa (Figure 3A). Strikingly, zebra finch cells in both types of chimeras are much more sensitive than the chick cells to the endogenous morphogen (Figures 3B and 3C) (n = 8/8 for Figure 3B and n = 4/4 for Figure 3C). Thus, when GFP-expressing chick cells are engrafted in a zebra finch host neural tube, zebra finch cells further away from the ventral source of SHH upregulate expression of SHH target gene *NKX6.1*, while adjacent chick cells do not. In a reciprocal pattern, chick GFP cells express *PAX7*, a gene that is repressed by SHH, at ventral levels where Pax7 is fully repressed in adjacent zebra finch cells (Figure 3B). In reciprocal grafts, zebra finch GFP cells upregulate expression of *NKX6.1* while chick cells at a comparable dorsoventral position in the chimera do not. Conversely, the grafted GFP-labeled zebra finch cells repress Pax6 at dorsoventral levels where the neighboring chick cells express PAX6 (Figure 3C). Differential response is cell autonomous, since isolated single chick cells are seen to be less sensitive to morphogen than their immediate neighbors (Figure 3B, arrows). Thus, zebra finch neural tube cells appear to be cell-autonomously more sensitive to SHH than their chick counterparts. This potentially explains their quicker patterning in response to less SHH.

**Figure 4:**
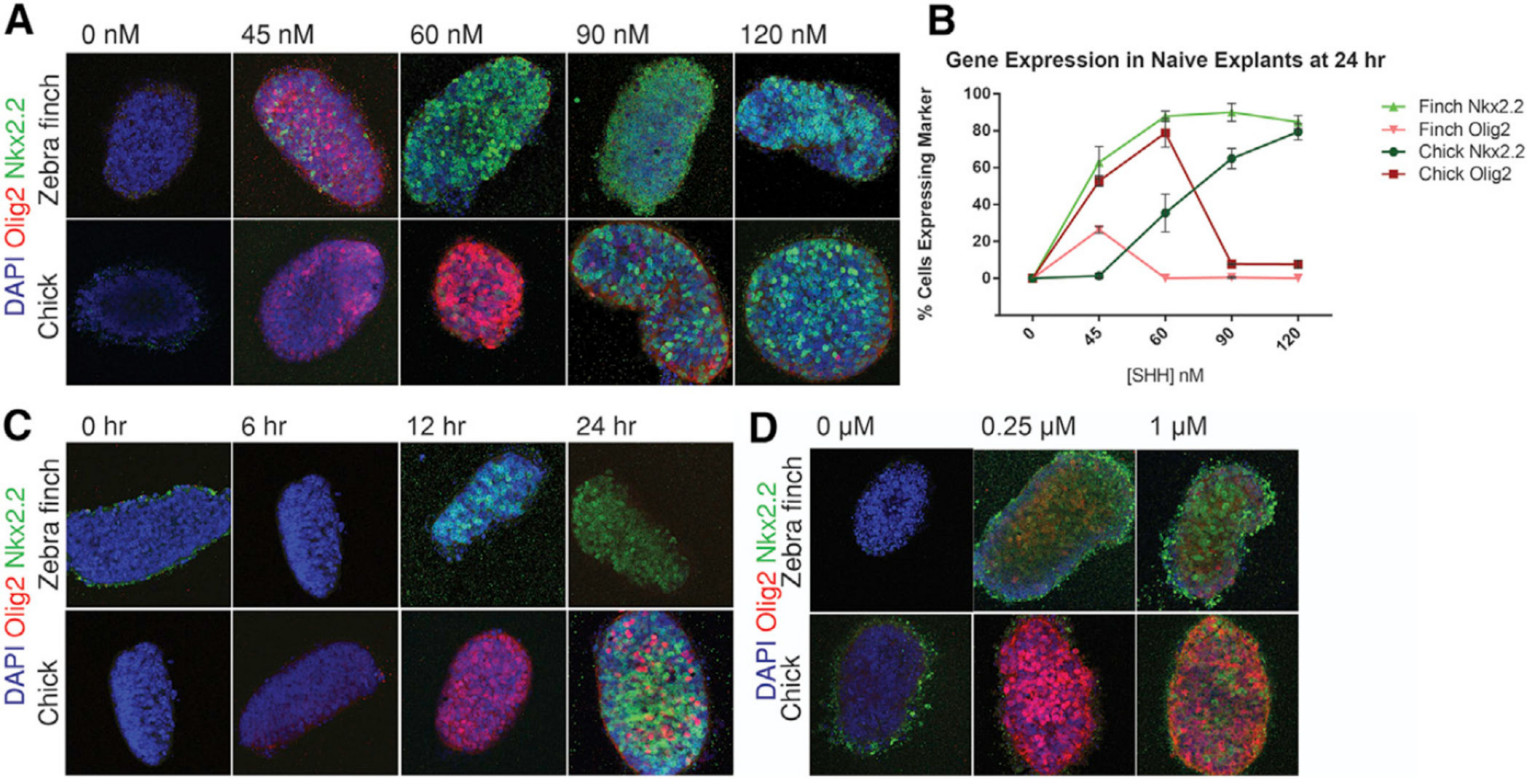
Intrinsic Differences in Morphogen Sensitivity Are Downstream of SMO (A) Different concentrations of recombinant SHH-N show that zebra finch and chick tissues have a differential response to the morphogen. Zebra finch cells upregulate the higher-threshold response gene *NKX2.2* at lower concentrations of the morphogen, compared with chick ex-plants, which still predominantly express the lower-threshold response gene *OLIG2* at similar concentrations (n ≥ 5/5 for each species and concentration). (B) OLIG2 and NKX2.2 expression on explants are quantified for different concentrations. Concentrations required for peak expression of both OLIG2 and NKX2.2 are lower for zebra finch explants. Error bars denote SD. (C) Differential response persists when explants are incubated at a fixed concentration of SHH (80 nM) but for different durations. Higher-threshold response genes are induced earlier in zebra finch explants compared with chick explants. n ≥ 3/3 for each species and concentration. (D) Cells retain differential sensitivity when the pathway is activated via SMOOTHENED directly. Intermediate naive explants were incubated for 24 hr in the presence of Smoothened agonist, SAG. Zebra finch cells are more sensitive to the lower and higher concentrations of SAG. n ≥ 3/3 each species and concentration.

To quantify differential responses of neural progenitors to SHH, we turned to in vitro explant assays. Naive intermediate [i] neural plate explants were isolated from zebra finch and chick embryos and incubated in vitro with different concentrations of recombinant SHH-N. After 24 hr, explants were harvested and immunostained for NKX2.2 and OLIG2 expression (Figure 4A). Low concentrations of SHH-N (45–60 nM) were sufficient to induce the high-threshold response gene NKX2.2 and low-threshold response gene OLIG2 in the finch explants, whereas the same concentration only induced expression of the low-threshold gene OLIG2 in chick explants. Concentrations of SHH required for peak expression at 24 hr were lower for both OLIG2 and NKX2.2 in the finch explants, compared with the peak expression in chick explants (Figure 4B, n ≥ 5/5 for each concentration of SHH-N in both species). Previous studies have shown that it is not only the absolute concentration of morphogen but also the duration of exposure that determines a cell’s response to SHH (Des-saud et al., 2007; Harfe et al., 2004). Therefore, we tested the response of tissues from the two species exposed to the same concentration of the morphogen, but for varying durations (Figure 4C). At 12 hr of exposure to a fixed concentration of 80 nM SHH, the zebra finch tissue was saturated for the high-threshold NKX2.2 response, whereas after the same duration of exposure the chick tissue only expressed the low-threshold OLIG2. At 24 hr, explants from both species were saturated for NKX2.2 (n ≥ 3/3 for each time point in both species). These results were confirmed by qRT-PCR (Figures S2A and S2B). Taken together, these results corroborate the conclusion that the zebra finch neural tube tissue is intrinsically more sensitive than the equivalent chick tissue to SHH. Lowering the thresholds at which target genes are differentially activated would contribute to the scaling of neural tube patterning in the smaller bird by allowing less SHH to achieve the same pattern of neural cell type specification.

### SHH Responsiveness Is Modulated through Differential Levels of Transcriptional Effectors

In principle, the intrinsic sensitivity to SHH could be modulated at a variety of levels, from receptor binding to signal transduction, or alternatively the effect of signaling could be modulated indirectly downstream of SHH target gene activation (Jessell, 2000). To identify the steps at which SHH sensitivity is altered between the chick and zebra finch, we first assayed activity of the Smoothened agonist (SAG) using [i] explants. SAG acts at the level of Smoothened (SMO), a transmembrane protein that initiates intra-cellular signaling. Similar to the differential response to SHH protein, zebra finch and chick cells demonstrated differential sensitivity to SAG (Figure 4D) (n ≥ 3/3 for each SAG), indicating that the mechanisms responsible for differences in morphogen sensitivity are intracellular and downstream of Smoothened.

**Figure 5:**
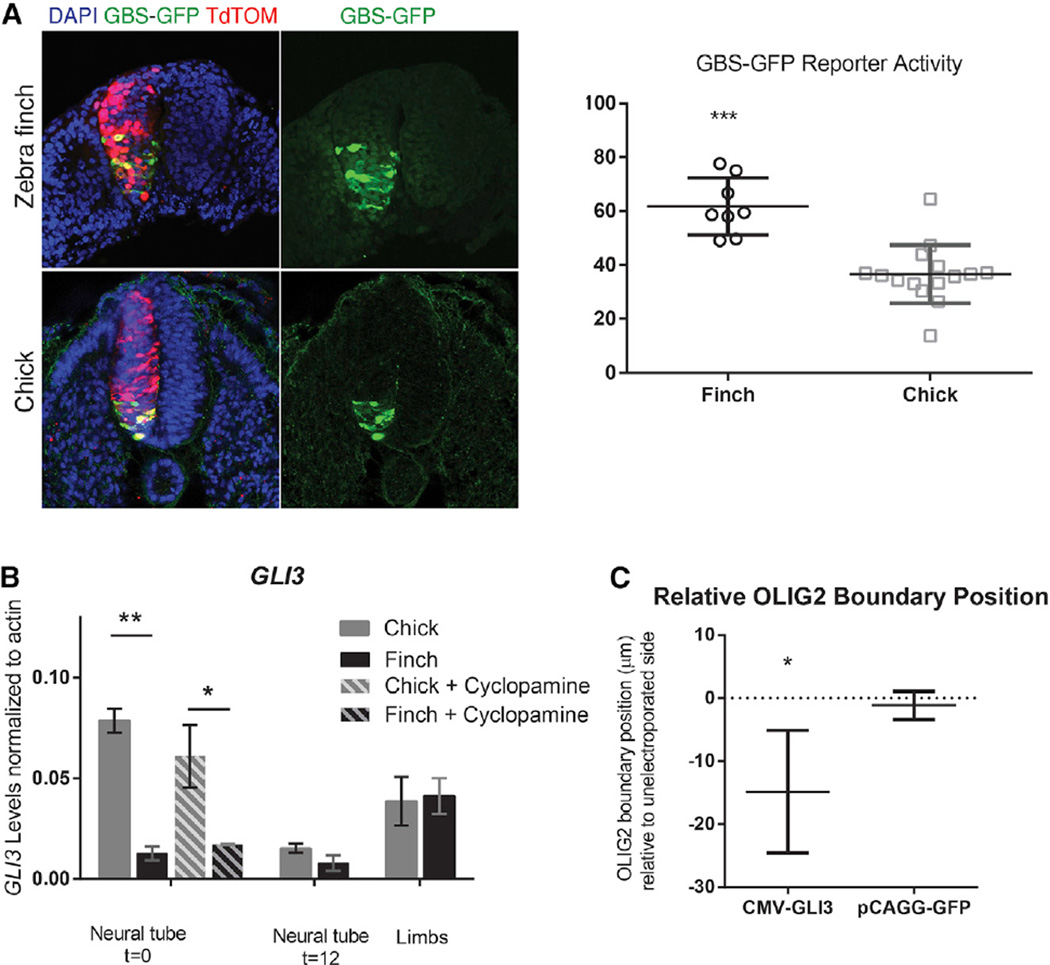
Differential Basal Levels of GLI3 in Zebra Finch Neural Tube Result in Differential Morphogen Sensitivity (A) Zebra finch and chick embryos were electroporated with a GLI activity reporter construct that contains eight consecutive repeats of GLI Binding Sequence driving the expression of GFP. Embryos were harvested 18 hr post electroporation and immunostained for GFP to assay the extent of reporter activity. Zebra finch neural tube had GLI activity observed up to 57% ± 12% of neural tube dorsoventral axis, whereas GLI activity was observed only up to 37% ± 11% (***p < 0.001). Error bars denote SD. (B) qRT-PCR on *GLI3* expression shows the levels are dramatically lower in the zebra finch naive neural tube explants, p < 0.005 at t = 0. After 12 hr of SHH exposure, *GLI3* levels are similar in chick and finch. GLI3 levels in stage HH20-21 level limbs is similar in chick and zebra finch, indicating that efficiency of species-specific primer amplification is comparable. To counter any previous SHH signal the naive tissues may have been exposed to, we injected cyclopamine into the neural tube and allowed it to incubate before collecting naive tissue. GLI3 levels were similarly lower in zebra finch naive explants. Error bars denote SEM.*p < 0.05, **p < 0.01. (C) St HH10-11 zebra finch embryos (15 hph) were electroporated with pCMV-GLI3-FLAG construct expressing full-length GLI3 at endogenous levels, or control pCAGG-GFP construct. When embryos are harvested 18 hr post electroporation and stained for OLIG2 expression, dorsal boundary positions for OLIG2 are different in the electroporated versus non-electroporated sides of the same embryo, indicating that response to SHH is downregulated in vivo. This is not observed in control electroporations.*p < 0.05.

We next asked whether the differential response is due to differences in the transmission of the signal between SMO and target gene regulation. To this end, we electroporated a GLI reporter construct (8xGBS-GFP) into zebra finch and chick neural tube at stage HH 10–11 (15 hph) and assayed reporter activity at 12 and 18 hr post electroporation (Figures 5A and S3A). At both time points, GLI activity in the zebra finch neural tube was observed more dorsally compared with that of chick. At 18 hr post electroporation, we observed GFP expression in cells in the ventral 37% (±11%) of the chick neural tube, whereas in the zebra finch embryos GFP expression reached up to 57% (±12%) of the distance to the dorsal midline of the neural tube (p < 0.001). Thus, the difference in sensitivity appears to lie somewhere in the SHH signal transduction cascade between SMO and target gene activation.

We examined the expression levels of various SHH signal transduction proteins in naive neural tissue from chick and zebra finch by RT-PCR. Both *PTC1* and *SMO* were expressed to equivalent levels in the two birds (Figures S2C and S2E). Similarly, the level of *GLI2*, the main activating transcription factor downstream of SHH in the neural tube, was equivalent in the two species (Figure S2D). By contrast, the expression of *GLI3*, the main repressive transcription factor downstream of SHH, was markedly lower in the zebra finch naive neural tube tissue than in chick (Figure 5B) (p < 0.005 for t = 0). To ensure the naive tissues were indeed not exposed to different SHH activity in different birds, we also incubated explants with cyclopamine in ovo, and saw a similarly significant difference in GLI3 levels. Importantly, no difference was observed in *GLI3* levels in limb bud tissue from the two species, verifying that the efficiency of the species-specific primer amplification is comparable in the two species and indicating that the differential regulation is neural tube specific (Figure 5B). We further verified these results utilizing single-molecule fluorescence in situ hybridization (FISH) as a second method to quantify GLI3 transcripts in the chick and zebra finch neural tube with species-specific probes (Figures S3B and S3C). GLI3 transcript levels were again seen to be distinctly lower in the zebra finch neural tube compared with the chick neural tube. Assessing GLI3 levels in the limb bud revealed that chick and the zebra finch had similar levels and distribution of GLI3 expression in this tissue, verifying that the assay was equally robust and equivalently sensitive in the two species.

It is known that SHH induces many target genes by derepression and that SHH activity achieves this primarily through depleting GLI3 levels (Oh et al., 2009; Persson et al., 2002). Thus a difference in the initial level of *GLI3* repressor could result in a change in sensitivity to SHH signaling. In a bird with less GLI3, it might take less SHH signaling to inactivate the repressor, hence making the tissue more sensitive. To confirm that levels of GLI3 can affect the dynamics of patterning in the neural tube, we artificially raised the GLI3 concentration in the zebra finch neural tube. As expected, electroporation-mediated expression of a pCMV-GLI3-FLAG construct encoding full-length GLI3 in stage HH 10–11 embryos (15 hph) downregulated the cellular response to SHH in vivo in zebra finch embryos (Figures 5C and S3D).

## DISCUSSION

Our study reveals a mechanism through which species that share a common body plan can adjust a morphogen-mediated patterning process to accommodate differences in size and achieve similar morphological proportions. We took advantage of the vertebrate neural tube as an established model system, whereby the readout for cellular morphogenic activity can be quantified, to identify a mechanism of pattern scaling downstream of SHH at the level of the cell-autonomous response to the signal. This conclusion contrasts with previous experimental and theoretical analyses that have focused exclusively on ligand concentration, diffusion, and opposing gradients, factors that are extrinsic to the cells (Ben-Zvi and Barkai, 2010; Ben-Zvi et al., 2014; Hamaratoglu et al., 2011; Houchmandzadeh et al., 2005). Our observations do not rule out a contribution of these other mechanisms of achieving scaling, in this or other systems; but do provide the first evidence of a cell-intrinsic contribution to the scaling of pattern to size.

A century ago, in his work *On Growth and Form*, biologist and mathematician D’Arcy Thompson wrote, “Like any other aspect of form, pattern is correlated with growth, and even determined by it” (Thompson, 1917). This principle greatly complicates the process required to achieve scaling. Not only does patterning take place over time, but the target tissues grow over time. Moreover, different species grow at different rates in addition to having different dimensions. One solution to this is to have the key steps of patterning take place in a short time window and at a time when embryos of different species have areas of similar size. Subsequent scaling can then be achieved by modulation of proliferation or differentiation rates. Indeed, such a differentiation-based mechanism is employed in the neural tube to accommodate both intra- and interspecific scaling in mice and chickens without requiring changes in morphogen gradient dynamics or response (Kicheva et al., 2014). However, this mechanism requires similar patterning dynamics and similar initial tissue size. We explored scaling of the ventral neural tube between different avian species, the zebra finch and the chick. We observed that ventral neural tube patterning in the zebra finch occurs on a smaller scale and over a shorter time period. These dynamic differences (more rapid patterning on a smaller scale) are accommodated, at least in part, through altering the sensitivity to the morphogen SHH in a cell-intrinsic manner.

The problem of scaling pattern in the neural tube between the zebra finch and chick is more complicated than this, however, as dorsal cell types also need to be scaled appropriately. These are patterned through a complementary gradient of BMP signaling emanating from the roof plate and surface ectoderm. While scaling of BMP activity has not been investigated in the context of the neural tube, it has been examined during dorsoventral patterning of the early *Xenopus* embryo. In this setting, pattern is adjusted relative to size through an expansion-repression mechanism. The BMP concentration gradient is established in a shuttling mechanism involving the BMP antagonist chordin. The shuttling, in turn, is limited either through a feedback loop with the chordin-proteinase inhibitor sizzled (Inomata et al., 2013), the BMP ligand ADMP (Ben-Zvi et al., 2008), or perhaps both working in concert (Inomata et al., 2013; Ben-Zvi et al., 2014). There is a similar need to scale BMP activity in the dorsal neural tube and, while distinct molecules may be involved, the logic is likely to be similar. These are mechanisms distinct from the scaling of SHH responsiveness described here, yet both processes must be active at the same time with the naive neural tissue. Moreover, the scaling of these two gradients must be integrated with one another to assure that the two morphogens scale proportionally.

A number of previous studies have underlined the importance of opposing gradients in size perturbations to the embryo, where antagonistic gradients from opposite poles can induce a self-regulating morphogenetic field (McHale et al., 2006; Plouhinec et al., 2013; Reversade and De Robertis, 2005). It is thus plausible that in the vertebrate neural tube a crosstalk between ventral SHH morphogenic field and dorsal BMP or WNT ligands emanating from the roof plate and surrounding ectoderm serves to fine-tune domain boundaries in proportion to overall size. Consistent with this idea, it was previously reported that in naive neural tube explants cultured in the presence of both SHH and BMP ligands, cells are induced to more dorsal cell fates in a dose-dependent manner compared with explants incubated with SHH alone (Liem et al., 2000). Moreover, WNT pathway ligands WNT1 and WNT3a secreted from the dorsal roof plate have been shown to oppose SHH activity through restricting *GLI3* expression (Alvarez-Medina et al., 2008; Yu et al., 2008). Thus, while our chimeric embryo experiments suggest that an intrinsic difference in SHH responsiveness persists in zebra finch versus chick cells that are exposed to the same levels of dorsally secreted BMP and WNT ligands throughout development, it is likely that opposing gradients additionally play a role as a fine-tuning mechanism for integrating scaling in the dorsal and ventral neural tube.

It is particularly interesting that the integration of the dorsal WNT activity gradient and the ventral SHH gradient has been tied to regulation of GLI3 transcription, as we have identified differences in GLI3 transcript levels as a mechanism for altering SHH sensitivity in different-sized avian neural tubes. Thus similar mechanisms appear to be exploited to scale patterns between patterns and refine patterns within a species. We found this difference to be specific to the neural tube, as transcript levels in the limb bud appear to be comparable between zebra finch and chick. It is likely that a different mechanism is required to scale SHH activity in the limb buds as ubiquitously lower levels of GLI3 in the zebra finch limb bud would be expected to result in patterning defects in the limb, as was previously shown in the extra-toes (Xt^j^/Xt^j^) background mice that are essentially GLI3 null (Litingtung et al., 2002). An attractive model for modulating GLI3 levels specifically in the neural tube and not in the limb bud would be through a pan-neural gene-regulatory mechanism. In future studies, it would also be interesting to look into post-translational modifications of GLI3 protein in the two species, if tools are developed to explore this in situ, as these could also contribute to the relevant levels of active GLI3 protein. However it is accomplished, our data indicate that differential basal levels of GLI3 result in differences in the sensitivity of the naive neural tissue to subsequent exposure to SHH. In turn, changing the sensitivity of neural tube cells to SHH allows a similar pattern to be established in smaller birds, making less SHH, as in larger birds producing more of the morphogen.

## EXPERIMENTAL PROCEDURES

For full details see Supplemental Experimental Procedures.

### Embryos and Embryonic Staging

All embryo experiments were performed in accordance with protocols approved by Harvard Medical School. Finch eggs and GFP finch eggs were obtained from Dr. Tim Gardner at Boston University, chick eggs were obtained from commercial sources (Charles River), and Roslin GFP chick eggs were obtained from Susan Chapman at Clemson University, with original work from Helen Sang (McGrew et al., 2008). All eggs were incubated at 38°C and embryos were staged with reference to HH staging series for chick and zebra finch staging series (Hamburger and Hamilton, 1992; Murray et al., 2013).

### Immunohistochemistry and Imaging

Embryos were fixed in 4% paraformaldehyde (PFA) at 4°C for 1 hr for stages up to HH 20, and 2–3 hr for older stages. After PBS washes, embryos were incubated in 15% sucrose overnight at 4°C. Next day, samples were embedded in 7.5% gelatin/15% sucrose/PBS, flash-frozen in cold isopentane, and cryosectioned at 14 µm. See Supplemental Experimental Procedures for the list of antibodies used for neural tube staining. Imaging was performed using a Zeiss confocal microscope at 40× (patterning), 63× (SHH gradient intensity analysis), and 100× (GLI3 RNA FISH). Images were analyzed with NIH ImageJ.

### Quantification of SHH Gradient in the Neural Tube

Chick and zebra finch neural tube sections were immunostained with the protocol described above, using Developmental Studies Hybridoma Bank 5 × 10^1^ antibody for SHH at a concentration of 1:20 in all samples. Imaging was performed using a Zeiss confocal microscope at 63× and analyzed with NIH ImageJ. For quantification of signal intensity, rectangular regions with width-spanning basal boundaries of the neural tube and dorsoventral height of 120 µm were positioned. The mean fluorescent intensity per pixel across the width as a function of height was calculated using the Plot Profile plugin function in ImageJ. To normalize the signal intensity to the background in each separate image, we calculated the background with similar methods as a mean value, across the width of a small rectangular region on the neural tube where no SHH is detected and all signal is assumed to be background. This value was subtracted from the data when plotting. Intensity graphs were generated using these values in GraphPad software. Data are the mean of n ≥ 3 for each stage represented.

### Chimera Transplants

To generate embryos with chimeric neural tubes, we dissected GFP chick donor and finch host, or GFP finch donor and chick host embryos were dissected in Tyrode’s saline (Voiculescu et al., 2008) at 12 hr of development (stage HH 3). Part of the tip of the Hensen node was transplanted from the donor into the host embryo, as described previously (Selleck and Stern, 1992). Embryos were placed on stretched-out vitelline membranes, and incubated on petri dishes with albumin for 24 hr in a humidified chamber at 38°C. They were then fixed in 4% PFA, embedded in gelatin, and cryosectioned at 12–14 µm.

### Naive Neural Plate Explant Surgery

Neural plate tissue was isolated from 10–13 somite stage (HH 10–11, or 15 hph) chick and zebra finch embryos as described previously in chick (Yamada et al., 1993). Recombinant mouse Shh-N from R&D Systems C25II (464-SH-025) and SAG (Millipore, 364590-63-6) were dissolved as instructed and added to the medium. When harvested (t = 0, 6, 8, 12, or 24), explants were either processed for qRT-PCR or immunostained as described.

### Single-Molecule Fluorescence In Situ

Chick and zebra finch neural tube from stage HH 11 embryos were collected and fixed in 4% PFA at 4°C for 1 hr, limb buds from both species were fixed in 4% PFA at 4°C for 2 hr, and all samples then were taken through PBS rinse, incubation in 30% sucrose overnight, and embedding in optimal cutting temperature compound. 10-µm-thick cryosections were collected for single-molecule FISH. Single-molecule FISH experiments were performed as described previously (Raj et al., 2008). For a more detailed explanation of sample processing and probe design, see Supplemental Experimental Procedures.

### Statistical Methods

GraphPad software was used to perform statistical tests and quantify significance. For analysis of transcription dorsal boundaries and progenitor numbers across developmental time points in chick versus zebra finch, multiple t tests were performed for each time point, as is listed on the graphs. For all analysis as depicted in the figures, significance is indicated as follows: *p < 0.05, **p < 0.01, ***p < 0.001.

## Supplementary Material

supp1

## Highlights

- Neural tube patterning is scaled to size in bigger chick and smaller finch embryos
- Chick and finch cells respond differently to SHH due to differences in GLI activity
- Scaling is achieved through cell-autonomous differences in morphogen sensitivity

## In Brief

Uygur et al. explore the problem of scaling anatomical patterns to variations in size during development, using the SHH-mediated patterning of the neural tube. They report a cell-intrinsic difference in morphogen responsiveness to be responsible for scaling pattern to size in different species, providing an alternative mechanism to the scaling of the morphogen gradient itself.
