# Supplementary material for: Scaling Pattern to Variations in Size during Development of the Vertebrate Neural Tube
Source: Dev Cell. Author manuscript; Available in PMC 2017 Jan 23. (PMC4854284; doi:10.1016/j.devcel.2016.03.024)
Supplement: supp1 [file NIHMS773159-supplement-supp1.pdf]

## Supplemental Information:

**Supplemental Figure Legends Figures S1-3**

**Figure S1. Assessment of patterning in chick vs zebra finch by number of progenitors. Related to Figure 1.** **A.** Number of progenitors expressing OLIG2 and NKX2.2 accelerates more rapidly in the zebra finch neural tube, compared to the chick. This implies that it is not just the progenitor domain boundary positions that progresses faster in the zebra finch, but also the number of progenitors that start expressing distinct transcription factors. Patterning occurs faster in the zebra finch overall. * *p<0.05,* ** *p<0.01,* *** *p< 0.001 .* **B.** Size of D-V axis is greater in chick than zebra finch in terms of length of D-V axis, as well as number of cells as shown in Fig.1D. Patterning appears complete in chick at 45 hph (gray asterisk) and 33 hph in zebra finch (black asterisk).

Supplementary Data Fig 1

A

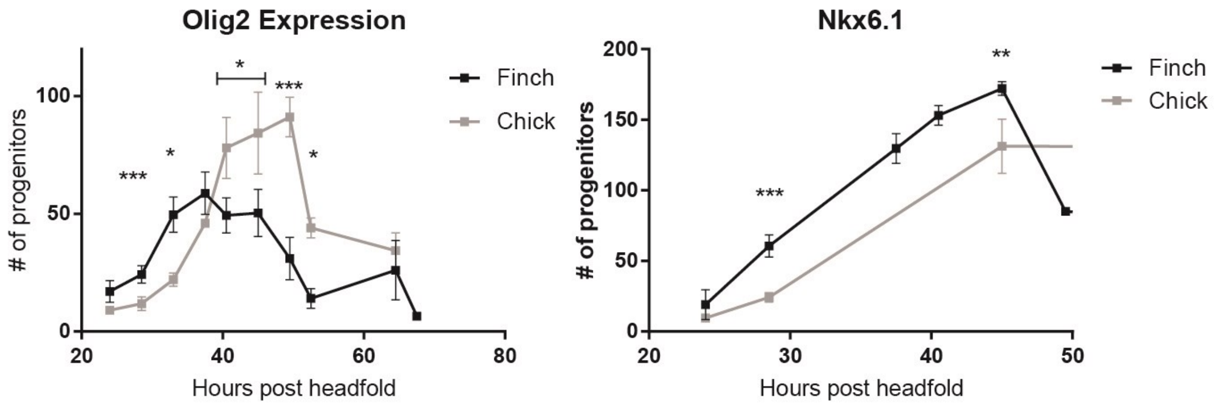


B

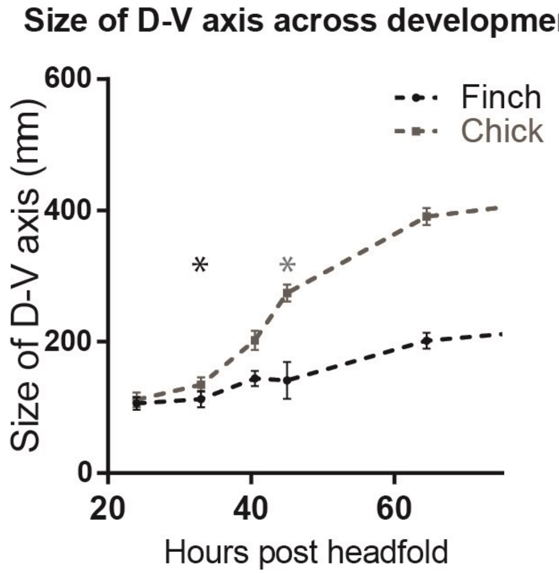


**Figure S2. Expression levels of distinct transcription factors and SHH pathway genes. Related to Figure 4.** Expression of SHH-induced transcription factors marks response to the morphogen in naïve neural tube [i] explants.

When levels of *OLIG2* (**A**) and *NKX2.2* (**B**) are assayed with qRT-PCR after incubation with SHH, and transcript levels are normalized to actin, we see that their expression peaks earlier in the zebra finch. This validates what we observe with explant immunostainings in Fig. 4B. **C**, **D.** We assayed levels of SHH pathway genes in naïve explants and explants incubated with SHH-N. Levels of *SMO* (**C**) and *GLI2* (**D**) did not appear to be different in the two species. **E.** PTC1 upregulation upon exposure to SHH also shows a similar trend.

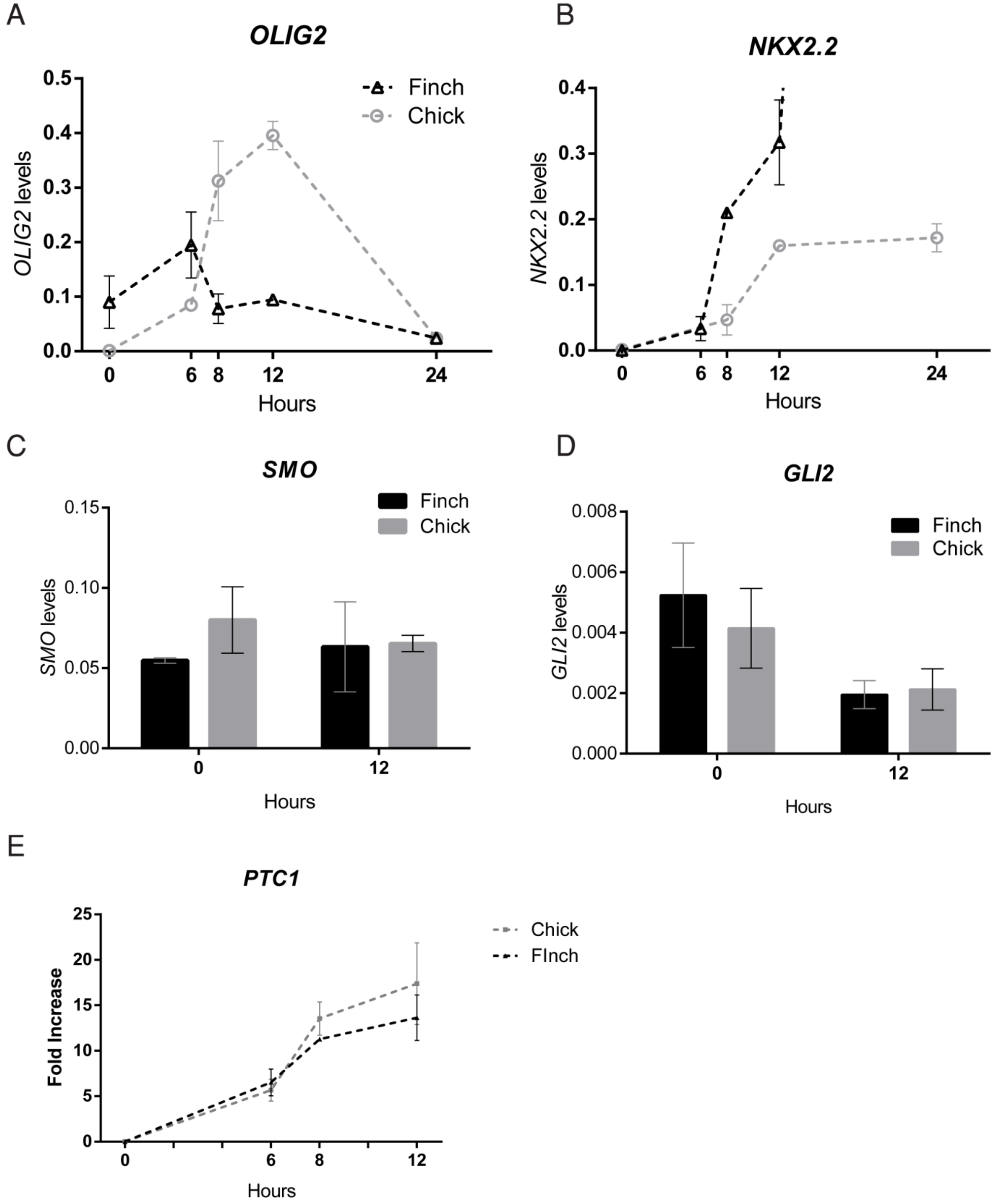

**Figure S3. Zebra finch has higher GLI activity in the neural tube due to lower levels of GLI3 expression. Related to Figure 5. A.** Both zebra finch and chick embryos were electroporated at stage HH10-11 (15 hph) with a 8XGBS-GFP construct and harvested at 12 and 18 hrs later. Reporter activity (GFP expression) extends more dorsally in the zebra finch neural tube at both timepoints, as quantified in Fig. 5A. Images representative for results in zebra finch and chick, n≥8 for species. **B.** Levels of GLI3 in the two species were tested with small molecule RNA fluorescent in situ hybridization (FISH) with species specific probes. GLI3 mRNA levels along the neural tube are different in the two species, as shown in panels i and iv. For better visualization, panels ii and v show close-up of an intermediate section of the finch and chick neural tube (marked in yellow rectangle in panels I and iv). GLI3 levels are comparable in the limb buds of two species, indicating that the difference is not due to detection or other technical issues. Data is quantified in in (**C**), n=5 for chick and 4 for zebra finch samples, at least 3 sections per samples are included in quantification. **D.** When electroporated with GLI3 plasmid with CMV promoter, zebra finch neural tube patterning is disrupted and progenitor boundary for OLIG2 is more ventral, compared with a control plasmid.

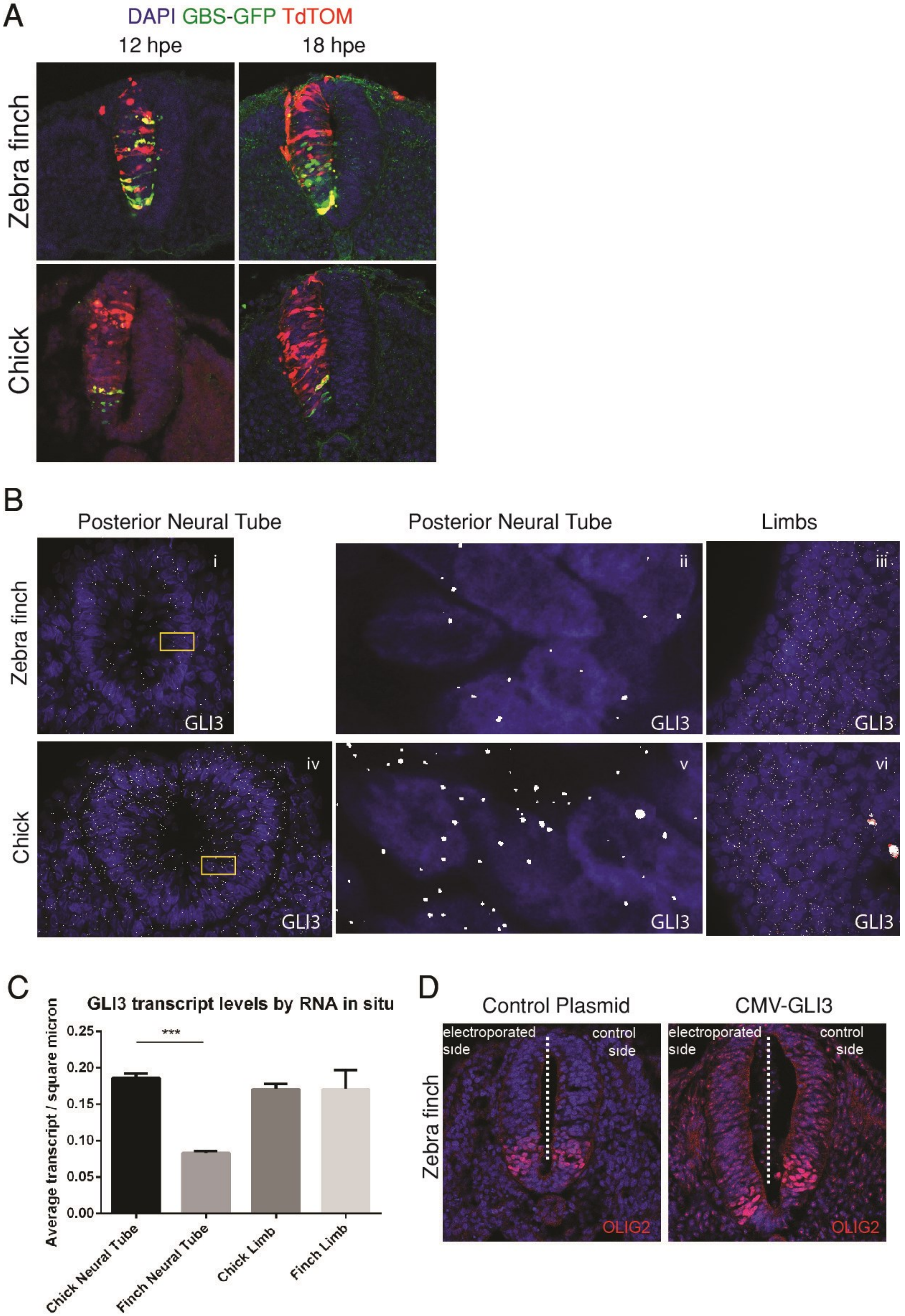
A
DAPI GBS-GFP TdTOM
12 hpe
18 hpe
Zebra finch
Chick
B
Posterior Neural Tube
Posterior Neural Tube
Limbs
Zebra finch
Chick
i
ii
iii
iv
v
vi
GLI3
C
GLI3 transcript levels by RNA in situ
Average transcript / square micron
0.25
0.20
0.15
0.10
0.05
0.00
***
Chick Neural Tube
Finch Neural Tube
Chick Limb
Finch Limb
D
Control Plasmid
CMV-GLI3
electroporated side
control side
Zebra finch
OLIG2

**Extended Experimental Procedures**

*Emrbyos and Embryonic staging*
Finch eggs and GFP-Finch eggs were obtained from Dr. Tim Gardner at Boston University, Chick eggs were obtained from commercial sources (Charles River, MA), Roslin GFP-Chick eggs were obtained from Susan Chapman at Clemson University, with original work from Helen Sang(McGrew et al., 2008). All eggs were incubated at 38°C and embryos were staged with reference to Hamburger Hamilton staging series for chick and zebra finch staging series(Hamburger & Hamilton, 1992; Murray, Varian-Ramos, Welch, & Saha, 2013). For comparing growth and pattern in earlier stages, we set somite number as the main reference point. To stay consistent across antero-posterior level and across species, all measurements were made at the level of somite 15, which is forelimb level in both species. As somite formation was 1.5 hr per somite in both species, we calculated developmental time as 1.5 hr x number of somites, and used this as time after headfold formation.

*Immunohistochemistry and imaging*
Embryos were fixed in 4%PFA at 4 °C for 1 hr for stages up to HH 20, and 2-3 hrs for older stages. After 3 times 5 minute PBS washes, embryos were incubated in 15% sucrose overnight at 4 °C. Next day, samples were embedded in 7.5% gelatin/ 15%sucrose/PBS, flash frozen in cold isopentane and cryosectioned at 14 um.
For immunostaining, gelatin was cleared from slides by incubation at 42 °C waterbath for 3 x 5 min. Blocking solution (1%BSA in PBS 0.1% Triton) for 1 hr, primary antibody (in blocking solution) overnight at 4 °C and secondary antibody (in blocking solution) for 2 hr at room temperature were performed. List of antibodies used for neural tube staining are as shown below. Imaging was performed using Zeiss Confocal and analyzed with NIH ImageJ.

| Antibody | From | Cat. # | Concentration |
|---|---|---|---|
| Nkx 2.2 | DSHB | 74.5A5 | 1:25 |
| Olig 2 | Millipore | AB9610 | 1:500 |
| Nkx6.1 | DSHB | F55A10 | 1:1000 |
| Pax 6 | DSHB | PAX6 | 1:40 |
| Pax 7 | DSHB | PAX7 | 1:20 |
| GFP | Millipore | AB16901 | 1:1500 |
| Shh | DSHB | 5E1 | 1:20 |

*Naïve neural plate explant surgery*
Neural plate tissue was isolated from 10-13 somite stage (St. HH10-11, or 15 hours post headfold) Chick and Zebra Finch embryos as described previously in chick(Yamada, Pfaff, Edlund, & Jessell, 1993). Recombinant Mouse Shh-N from R&D Systems C25II (464-SH-025) and SAG (Millipore, 364590-63-6) were dissolved as instructed and added to the medium. When harvested (t= 0,6,8,12 or 24) explants were either processed for qRT-pcr or immunostained as described. For samples with cyclopamine incubation, we injected cyclopamine in ovo at stage HH9 and harvested naive explants after 10-12 hours of incubation.

*qRT-PCR on explants*
For each data point, 15-25 naive explants were isolated, only 2-4 explants per embryo. Explants were embedded in collagen (PureCol, Advanced Biomatrix), incubated at 37 °C for 35 min and dissociated with extraction buffer (Buffer XB) in Arcturus PicoPure RNA Extraction kit (Life Technologies, KIT0204). RNA isolation was performed as described in kit protocol. Immediately following RNA extraction, cDNA was made using Superscript II (Life Technologies,18064-014). Standard curves (with 8 serial dilutions) for each set of species specific primers was performed to accurately assess expression levels. Levels were normalized to actin. Primer sequences for each gene are as follows:

| Gene | Species | FW primer | REV primer |
|---|---|---|---|
| *ACTIN* | Finch | GTGCGTGACATCAAGGAGAAG | CCTGAACCTCTCATTGCCA |
| | Chick | GTGCGTGACATCAAGGAGAAG | CCTGAACCTCTCATTGCCA |
| | Finch | GCGCGAGATCGTGCGCGACA | TCTTCTCCAGCGAGGAGGAG |
| | Chick | GAGGGAGATCGTCCGTGATA | TTTTCTCCAGGGAGGAGGAT |
| *PTC1* | Finch | AAGCGAACAGGAGCAAGTGT | TGCTGCTTGGAGTGAAAATG |
| | Chick | AAGCGAACAGGAGCAAGTGT | TGCTGCTTGGAGTGAAAATG |
| *SMO* | Finch | TTTGTCATGCTCACCTATGC | ACCAGGTGATGAGGTGGAAG |
| | Chick | TTTGTCATGCTGACCTACGC | ACCAGGTGATGAGGTGGAAG |
| *OLIG2* | Finch | GGATGCACGACCTCAACC | CTTCATCTCCTCCAGGGAGT |
| | Chick | GGATGCACGACCTCAACA | CTTCATCTCCTCCAGCGAGT |
| *NKX2.2* | Finch | ACGCAGGTGAAGATCTGGTT | TTGTACTGCATGTGCTGCTG |
| | Chick | ACCTTCCAGACGGGCATC | TGTAATGGGCGTTGTATTGC |
| *GLI2* | Finch | GCAACTAAGGAAACACATGA | CCCATGAGCAGGAATCCTTA |
| | Chick | GCAGCTGAGGAAACACATGA | CCCATGAGCAGGAATCCTTA |
| *GLI3* | Finch | AGTCAGCTCTGGACCCTCTG | AGGTCCTCGTCAGGCTTTAT |
| | Chick | GGTCAGCTCTGGACCTTCTG | AGGTCCTCATCCGGCTTTAT |

*Electroporations*
8XGBS-GFP Gli reporter construct was co-electroporated into Chick and zebra finch neural tubes (along with pCAGG-TdTomato control construct) at stage HH10-11, samples were harvested at either 12 or 18 hours post electroporation. pCMV-FLAG-Gli3 or control constructs were electroporated into zebra finch neural tube at stage HH10-11, samples were harvested 18 hpe. All samples were fixed at 4%PFA and immunostained.

*Small molecule RNA Fluorescent In Situ*
Tissues from posterior neural tube of stage HH10 chick and zebra finch embryos were fixed in RNAse free conditions, 4% PFA for 1 hr on ice, taken through 30% sucrose solution in gradients and embedded in 100% OCT. Tissue was sectioned, and taken through PFA, PBS, 70% ethanol washes. Species specific fluorescent probes (48 probes for chick and zebra finch separately) were designed using the STELLARIS software. Single Molecule FISH was performed as previously described (Lyubimova et al., 2013), and sections were imaged on a DeltaVision microscope. For quantification, the neural tube in each section was divided along its dorsoventral length into thirds, and transcripts were counted in the middle 1/3rd of tube (i.e., the intermediate naive tissue) using ImageJ, normalized to the area of the region of interest, and averaged across left and right sides. A total of 5 chick and 4 finch neural tubes at stage HH10 were used for quantification with at least 3 separate sections counted per individual neural tube.

**Supplemental References**

Hamburger, V., & Hamilton, H. L. (1992). A series of normal stages in the development of the chick embryo. 1951. *Dev Dyn*, *195*(4), 231–272. doi:10.1002/aja.1001950404

Lyubimova, A., Itzkovitz, S., Junker, J. P., Fan, Z. P., Wu, X., & van Oudenaarden, A. (2013). Single-molecule mRNA detection and counting in mammalian tissue. *Nature Protocols*, *8*(9), 1743–1758. doi:10.1038/nprot.2013.109

McGrew, M. J., Sherman, A., Lillico, S. G., Ellard, F. M., Radcliffe, P. A., Gilhooley, H. J., … Sang, H. (2008). Localised axial progenitor cell populations in the avian tail bud are not committed to a posterior Hox identity. *Development (Cambridge, England)*, *135*(13), 2289–99. doi:10.1242/dev.022020

Murray, J. R., Varian-Ramos, C. W., Welch, Z. S., & Saha, M. S. (2013). Embryological staging of the Zebra Finch, Taeniopygia guttata. *Journal of Morphology*, *274*(10), 1090–110. doi:10.1002/jmor.20165

Yamada, T., Pfaff, S. L., Edlund, T., & Jessell, T. M. (1993). Control of cell pattern in the neural tube: motor neuron induction by diffusible factors from notochord and floor plate. *Cell*, *73*(4), 673–686. doi:0092-8674(93)90248-O [pii]
